# The Asian Cardiovascular and Thoracic Annals turns 30

**DOI:** 10.1177/02184923221145328

**Published:** 2022-12-21

**Authors:** Carlos A Mestres, Frank L Tamru, Arkalgud Sampath Kumar, Yutaka Okita

**Affiliations:** 1 Adult Cardiac Surgery, 37702 The University of the Free State, Bloemfontein, South Africa; 2 Mays Landing, NJ, USA; 3 Chief Cardiothoracic Centre, All India Institute of Medical Sciences, New Delhi, India; 4 Takatsuki General Hospital, Takatsuki, Osaka, Japan

**Keywords:** *Asian Cardiovascular and Thoracic Annals*, history, anniversary

## Abstract

*The Asian Cardiovascular and Thoracic Annals* turns 30 in 2023. A historical review since it was first published in March 1993 is presented.

‘The beginning is the most important part of the work’ – Plato

## Introduction

Out of economics, maturity is defined as the quality of behaving mentally and emotionally like an adult or as a very advanced or developed form or state.^1^ The same Cambridge Dictionary gives the example ‘it takes maturity to be leader’. Having said that, our publication, *The Asian Cardiovascular and Thoracic Annals* (from now on *Asian Annals*) the official journal of the *Asian Society of Cardiovascular and Thoracic Surgery* (ASCVTS) turns 30 in 2023. It was first launched in March 1993. These three decades have not been comfortable and the path was unevenly paved, considering the difficulties in starting a scientific journal from scratch which aimed at covering the needs of the largest region of the world, Asia and the Pacific, including Australia and New Zealand. Gaining credibility is not an easy task and it takes years to reach a certain level of quality that ensures a good flow of contributions to support the solidity of the given publication.

Thirty years after the first issue, it can be stated that *Asian Annals* has reached its maturity. A number of changes were implemented since its start and now is the time to sit down and reflect on what has been done and what we imagine the future of *Asian Annals* will be. This look at the future has been recently addressed by the editors led by the Editor-in-Chief, Dr Yutaka Okita in a position statement that aimed at defining our goals and the expected quality.^2^

The following is a summary of these first thirty years of life of *Asian Annals*. This is an important event, an anniversary to remember, for the readership to incorporate in full the exact meaning of the words.^1^ Then, *Asian Annals* is now a leading publication in cardiothoracic medicine and surgery in Asia.

## The mission and the goal

Phrased in slightly different ways over the years, wording always counts to express feelings, to reach the readership positively. Our initial issue confirmed that our mission was and it is to provide an international forum focusing on topics of interest to the Asia Pacific region, emphasizing issues affecting our patient population and the medical professionals and facilities that serve them.^3^ The mission did not change; however, the message was conveyed according to times and after its launch, it was expressed as providing a continuing forum whereby clinicians from all cardiac communities in Asia and the Pacific Rim can carefully review, interpret and guide the momentous forces of change influencing the delivery of quality, cost-effective healthcare to our population.^4^ A most recent wording confirms that our mission is to provide a forum for cardiovascular and thoracic surgeons, cardiologists and allied health care professionals from Asia and the Pacific Rim to discuss the diagnoses and treatment of cardiovascular and thoracic diseases from a regional perspective^5^ as one can access on the website. Different wording, same message, adapted to times.

The goal of *Asian Annals* has been to deliver to the region a journal of uncompromising professional and ethical standards. From the outset, *Asian Annals* endeavoured to document and integrate the development and advancement of Asian healthcare. Thirty years later, our humble progress indicates that our mission was appropriate and the goal achieved.^2^

## Asia Pacific Publishing Exchange (APEX)

**Figure 1.**
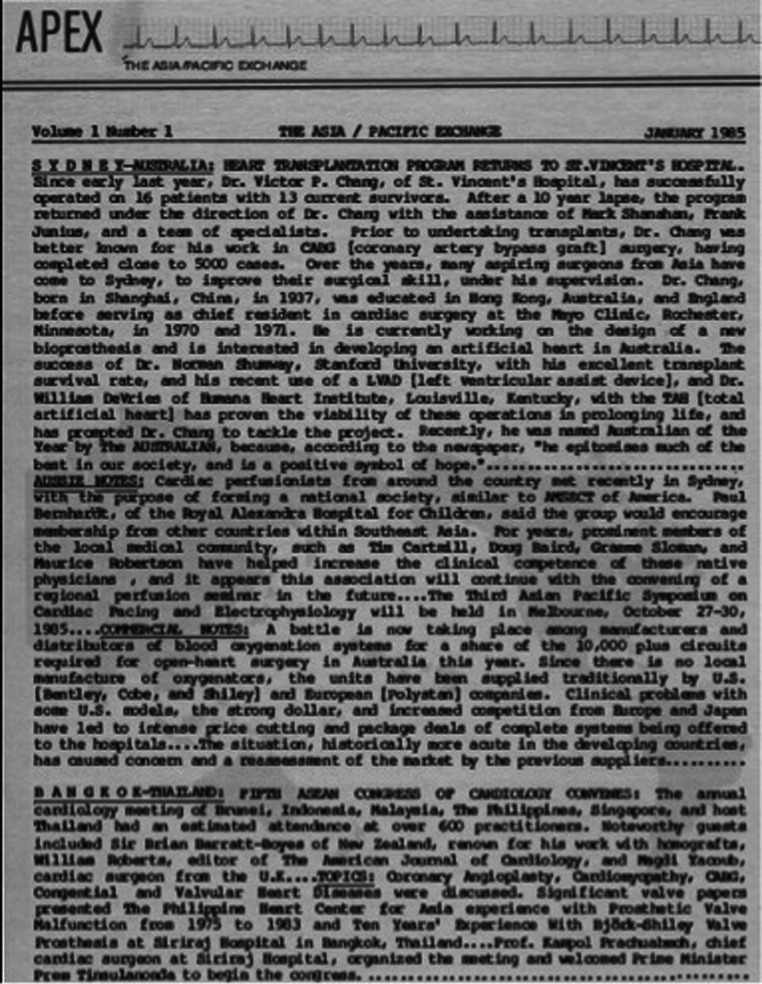
The first publication of Asia Publishing Exchange (APEX), the prelude for *Asian Cardiovascular and Thoracic Annals*, ‘A Cardiac Newsletter for Asia’.

Frank Tamru, the founder of APEX, a Hawaiian-based enterpreneur, felt the need for an Asian voice in cardiovascular and thoracic surgery. He launched the publishing company in 1984 from his home in Hawaii after four years of calling on heart surgeons in the Asia Pacific Region representing an American cardiac device manufacturer. APEX in 1986 was incorporated and headquartered in Singapore on Orchard Road, in the heart of downtown in Liat Towers. That first publication ‘A Cardiac Newsletter for Asia’ (Figure 1) in January 1985 served as a prelude for *Asian Annals* and continued to being published for eight years. After the idea was generated in 1991 to launch an Asian scientific journal for heart specialists, it took three years of intense fieldwork, usually on late evenings and over the weekends at an apartment at Leonie Hill, a residential neighbourhood of Singapore, for two of the authors (FLT and CAM) to design, model and develop which type of journal would suit the needs of the surgical community across Asia. A solid business model and a plan for financing the cost of the journal were established at that time.

Initially, to convene a credible editorial board, Tamru created the ACE (Asian Cardiovascular Exchange) group comprised of the current leading heart surgeons from Singapore, Malaysia, Indonesia, Japan, India, Thailand and Korea. He had previously represented Shiley Inc. throughout Asia and was quite familiar with a prestigious cohort of surgeons and cardiologists. All eagerly accepted to serve on the editorial board once the plan evolved further. The initial investment came from his industry contacts, early advertisers were confirmed and the journal was launched in March 1993.

## The voice of cardiothoracic medicine in Asia

A motto is a short sentence or phrase that expresses a belief or purpose.^1^ APEX was a consistent (13 issues between 1985 and 1992) and focused tool for communication for clinicians and the industry. Varied business and surgical updates, new device introductions and readers’ opinions were compiled in printed ‘melting pot’ housing news for the region. APEX became a timely and trustable vehicle that gained popularity over the years.^6^ However, there still was a gap to fill in the form of a credible journal. When *Asian Annals* was finally launched, an appealing motto ‘The Voice of Cardiothoracic Medicine in Asia’ was chosen as it perfectly matched the Mission of *Asian Annals*, to provide a forum for cardiovascular and thoracic surgeons, cardiologists and allied health care professionals from Asia and the Pacific Rim.^2^ This holds true today.

## The editors

The Editor-in-Chief (EIC) is the highest-ranking member of the Editorial Team of any given media and the individual with full responsibility for its operations and policies. The EIC is the final arbitrator of all decisions of a given publication, such as the decision to publish or reject a contribution, the publication's scope, inquiries and editorial priorities.^7^ These decisions are of critical importance for the quality and prestige of any publication, especially in current times where scientific and academic fraud are more common than expected.^8–11^ Among the different responsibilities of the EIC, one of the most important is to ensure that ethical standards are kept to the highest level. Furthermore, the EIC has to be instrumental in ensuring an appropriate relay when the term is over, something which might not be easy, as taking over from a predecessor implies a learning curve, too. This, without disturbing the smooth journal process.^12^

**Figure 2.**
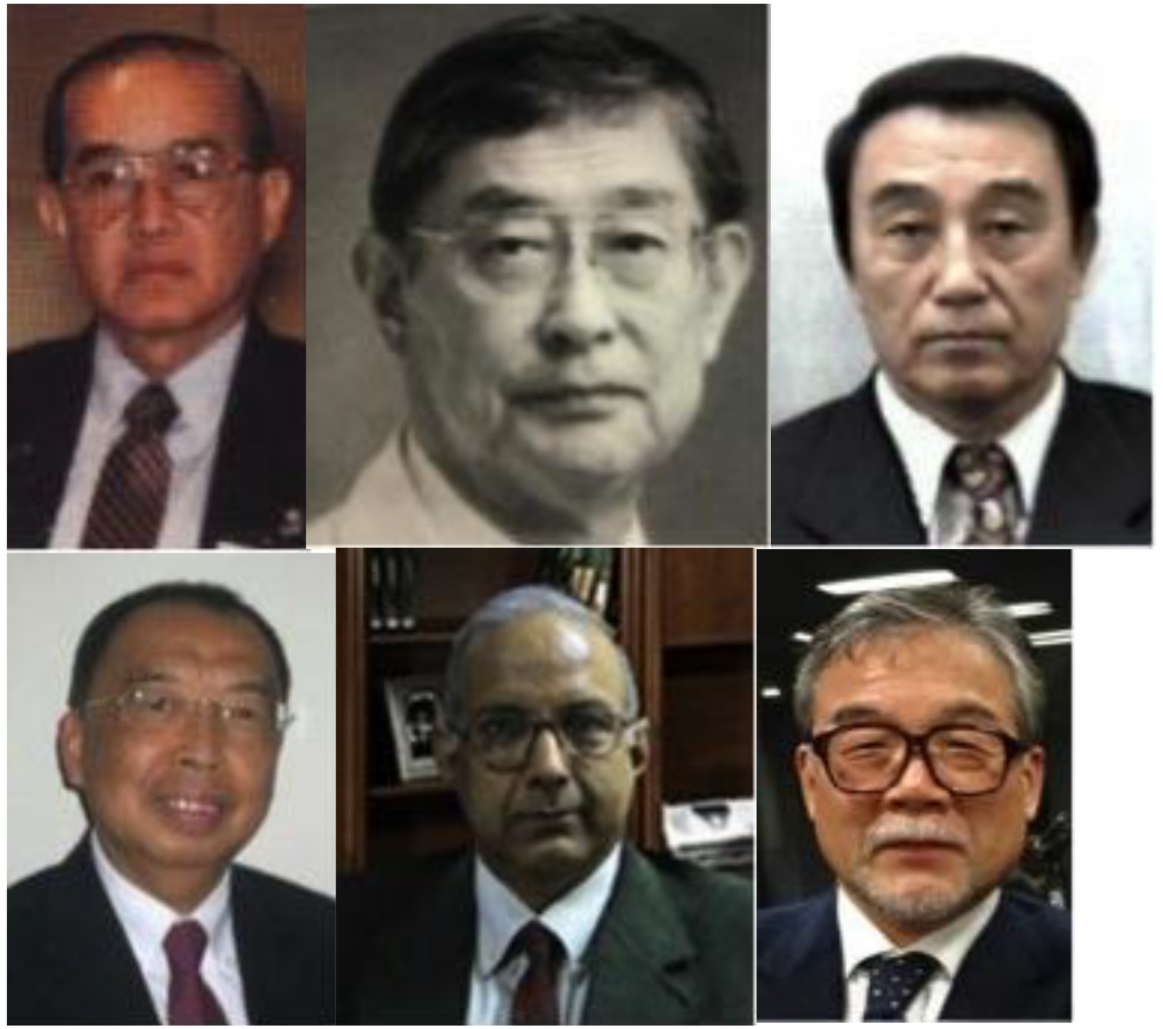
The editors of the *Asian Cardiovascular and Thoracic Annals*.

The first EIC, the late Dr Kampol Prachuabmoh from Bangkok (1927–2019) was chosen as he was the senior-most member of the ACE group and a respected colleague in Thailand and across Asia. He served as EIC between 1993 and 1994 and oversaw the birth of a truly Asian project which at that time had not a societal affiliation, a goal that took some time to be reached. These first few years were marked by the difficulties of starting a journal in the region as he highlighted in a compelling editorial^13^ (Figure 2).

Prof. Hitoshi Koyanagi from Tokyo was the EIC from 1995 to 1996. He was instrumental in accelerating the progress of *Asian Annals*, whose growth remained slow. A primary goal was to convince the Asian readership to capture their scientific production in a homegrown publication.

Dr Cho Bum Koo from Seoul took over in 1997; under his seven-year tutelage, *Asian Annals* expanded into the international arena and was included into the CTSNet journal collection thanks to the close relationship Publisher Tamru had with three influential western surgeons who were promoting CTS membership in Asia.^14^ Of interest, both Asian Associations, the Asian Society of Cardiovascular Surgery (ASCVS) and the Association of Cardiovascular and Thoracic Surgeons of Asia (ACVTSA), were simultaneously affiliated in different ways with *Asian Annals* for some time. The three first editors cooperated with the Editorial Office in Singapore led by Tamru's dedicated invaluable Managing Editor Mrs. Patricia Siow.

In 2004, Dr David LC Cheung from Hong Kong took over the reins from Dr Cho and relocated the Editorial Office to Hong Kong, where he oversaw *Asian Annals* under its renewed format. The editorial production was handed over from Asia Publishing Exchange Pte. Ltd to a new team in Hong Kong supervised by Mrs. Lindy Chan, working with SAGE Publications (currently SAGE Publishing), to reduce production costs. During his tenure, Dr Cheung oversaw the transition to electronic manuscript processing, completed a few years later. This favoured a faster review process and improved the efficiency of the editorial process. The number of issues increased from four to six yearly.^15^ Dr Cheung finally transferred *Asian Annals* to SAGE Publications.

Dr Arkalgud Sampathkumar's tenure started in 2011, the longest of any EIC of *Asian Annals* so far. Dr Sampathkumar from Delhi, a former EIC of the Indian *Journal of Thoracic and Cardiovascular Surgery*, choose three dedicated section editors to deal with adult cardiac surgery, congenital surgery and thoracic surgery. Although associate editors were assisting the EIC since 1993, this assignment by area has been active right up today. He brought in new members for the International Advisory Board and incorporated experienced reviewers.^16^ Under Dr Sampathkumar, who worked closely with SAGE's Mr Will Rushton, *Asian Annals* went to 9 issues a year, starting in 2014. Further, reciprocal ads in *Annals of Thoracic Surgery, the European Journal of Cardiothoracic Surgery* and the *Journal of Thoracic and Cardiovascular Surgery* were exchanged to expand the journal's visibility. In 2018, in a meeting of all the journal editors in Boston, the agreement was made to co-publish important articles on guidelines in all the journals. Additionally, *Asian Annals* joined the Cardiac Surgery Intersociety Alliance (CSIA). This is an initiative of the American Association for Thoracic Surgery (AATS), the Society of Thoracic Surgeons (STS), the European Association of Cardio-thoracic Surgery (EACTS), the Asian Society of Cardiovascular and Thoracic Surgery (ASCVTS) and the World Heart Federation (WHF). The CSIA began with a desire to assist underserved populations with cardiac surgery, particularly in Africa. The Memorandum of Understanding (MOU) is signed by all association presidents. Dr Sanghoon Jheon signed the agreement to support the CSIA in his function as President of ASCVTS.

Dr Yutaka Okita from Kobe, the current EIC, took over at the beginning of 2020. He has revamped the Editorial Board, incorporating a fourth section editor for aortic surgery. The International Advisory Board has also been renewed. Through these difficult and challenging times of the COVID-19 pandemic, Dr Okita has successfully led *Asian Annals* and started publishing Special Issues^17,18^ which, together with an increasing number of original and review articles, will hopefully reach a major goal of receiving an impact factor which has been preliminary calculated by SAGE Journals at 1.272.^2^

## The Editorial Board

The Editorial Board (EB) is a group of professionals with proven clinical and research experience and prominent people in the fields of interest of the journal. The EB is also known by other names like Advisory Board. The importance of the EB cannot be neglected, as this prestigious group is also a part of the Journal's corporate image. The members of the EB support the EIC in a number of different ways, from selecting reviewers to writing editorials or another kind of articles. The EB is well scrutinized by the readership. Hence, choosing the appropriate members is also an important task for the EIC that should positively revert to the journal.

**Figure 3.**
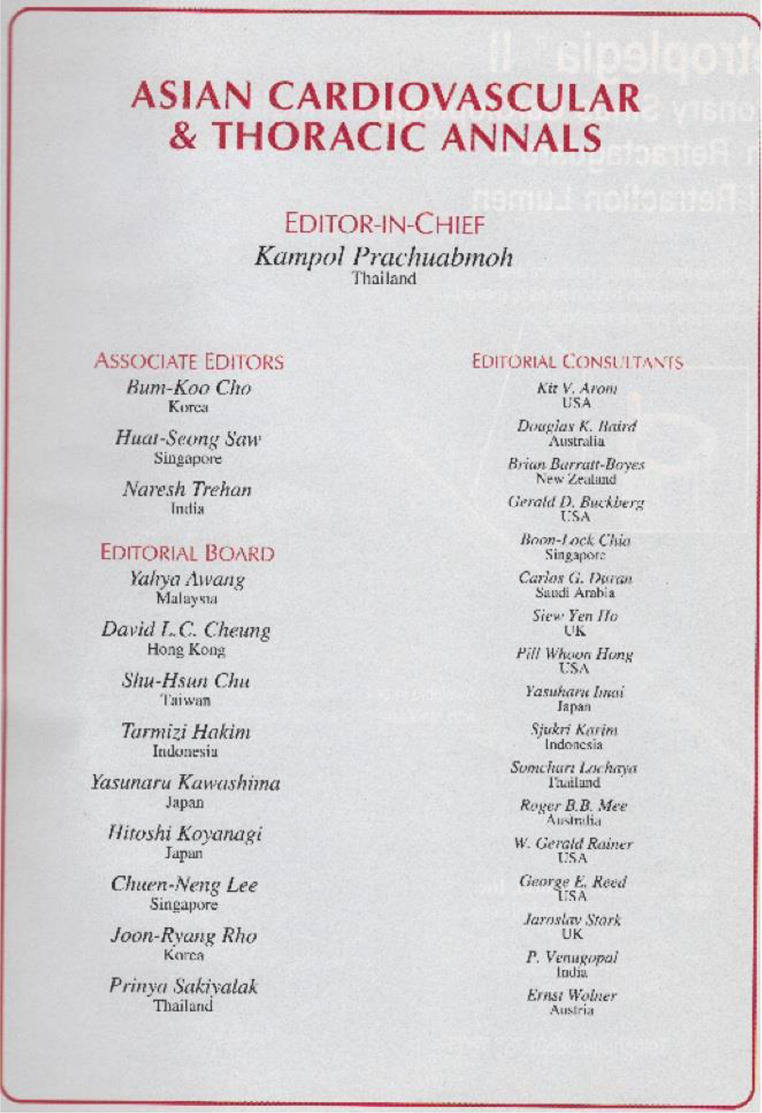
The first Editorial Board of the *Asian Cardiovascular and Thoracic Annals*.

The composition and number of members of the EB change from journal to journal. There are no specific rules as to how many members the EB should have. The journal has to consider expanding the EB and making it as international as possible, which increases the exposure of the EB across different regions. *Asian Annals* understood this from the outset. The initial *Asian Annals* Board included the first three Associate Editors, Drs. Cho Bum Koo from Seoul, Saw Huat Seong from Singapore and Naresh Trehan from Delhi (Figure 3). Tamru actively researched and confirmed 9 Editorial Board Members and 16 Editorial Consultants from 11 countries in consultation with Mestres and others to serve on the Board. Changing over time, the current Board includes four Lead Section editors (Thoracic, Adult Cardiac, Aortic, Pediatric/Congenital) assisted by 25 editors from 11 countries. In 2022, the International Advisory Board includes 14 members from seven countries. *Asian Annals* is, by definition, an international journal.

## The cover

The cover or front page of a given publication is what the reader first sees when holding it in hand or when watching it on the computer or at a handheld device. As such, the cover is used by the Publisher to gain the attention of readers. It becomes the face of a particular communication medium. Over the years, printed or digital media has invested time and, eventually, significant resources to improve covers. From no photographs to black and white images to color pictures, a variety of layouts and backgrounds have been used to capture the readership's attention.

Front pages have also inspired art in the form of animated scenery. An example is that of the New York Times. Artist Josh Begley compiled in an animation the evolution of The New York Times front page in a quickfire flip book showing all front pages since 1852.^19,20^ Another example is that of La Vanguardia, one of the oldest newspapers in Spain established in 1881, whose cover and format underwent a complete technological transformation in 1989 with a new design^21^ projected by the late Milton Glaser, the creator of the iconic ‘I Love New York’ logo, likely one of the most widely imitated images in history. Yomiuri Shimbun, one the most important newspapers in Japan, was first issued in 1874 and is credited with having the largest newspaper circulation in the world with over 9 million daily,^22^ launched in 1955, the Japan News, an English-language daily adopting its current name, layout and content in 2013 as a part of a major revamp.^23^

The same happened to the major journals in the field of Cardiothoracic Surgery, *The Journal of Thoracic and Cardiovascular Surgery*, *The Annals of Thoracic Surgery* and *The European Journal of Cardiothoracic Surgery*. They all have undergone several changes in structure and contents over the years, adapting to the observed trends and needs. Publishing is a dynamic process, and changes are a must. The major journals have revamped their covers, too, and different designs have been sequentially introduced and integrated by their readership. Their historical aspects can be formally checked on their websites and specific commemorative articles.^24–26^

**Figure 4.**
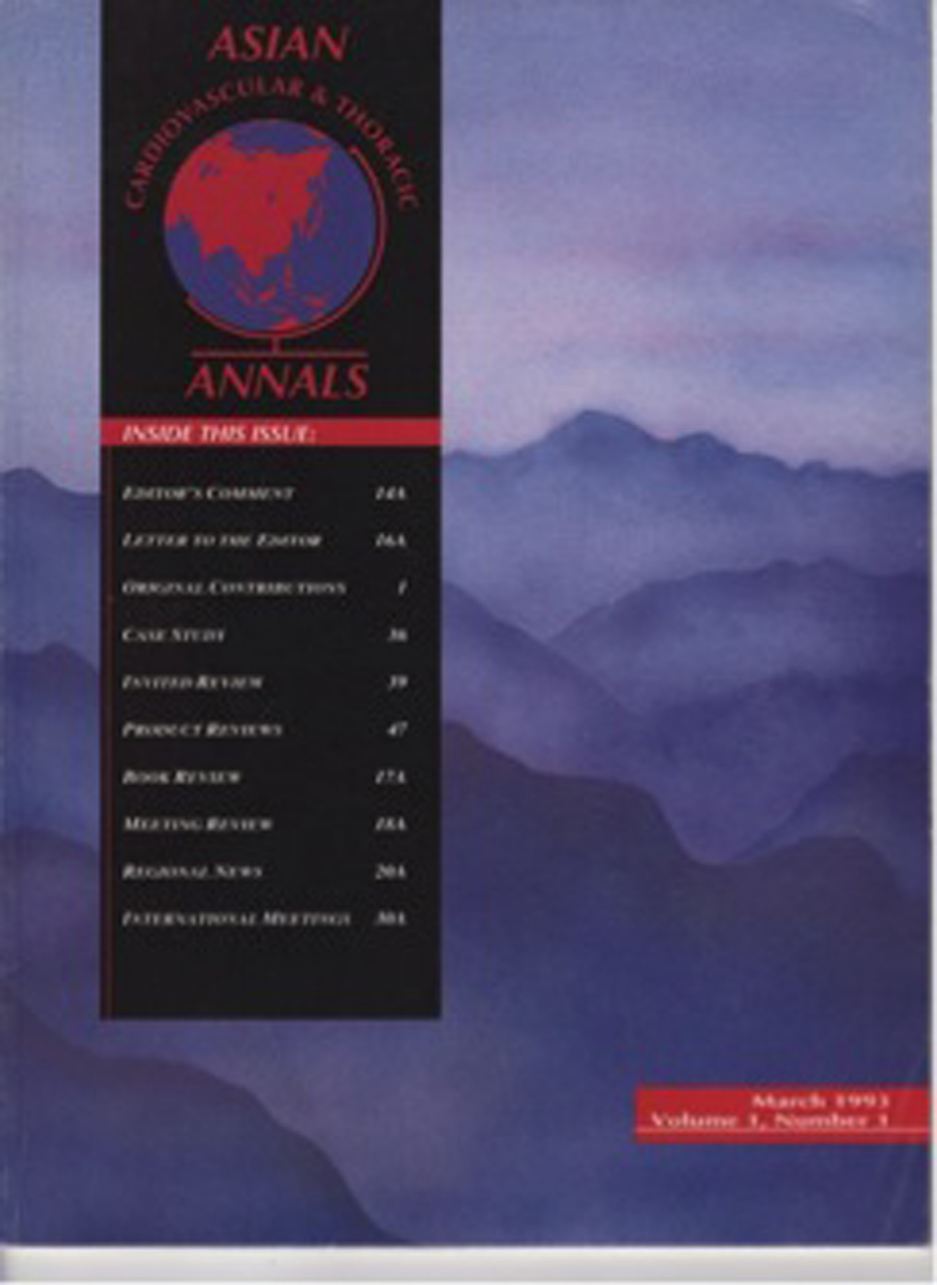
The cover of volume 1, number 1.

The Publisher knew the cover would be one of the most important components of *Asian Annals* once it was launched in 1993. Initially, it included a soft purple background and terrestrial globe displaying the vastness of Asia to its readership (Figure 4). This cover was awarded a design prize in the USA and was the ‘business card’ of *Asian Annals* for the first three years of operations. *Asian Annals* had a distinctive look and was published in cooperation with Silent Partners Inc. a publishing company from Austin, Texas, USA, managed by Mrs. Lori Brix.

**Figure 5.**
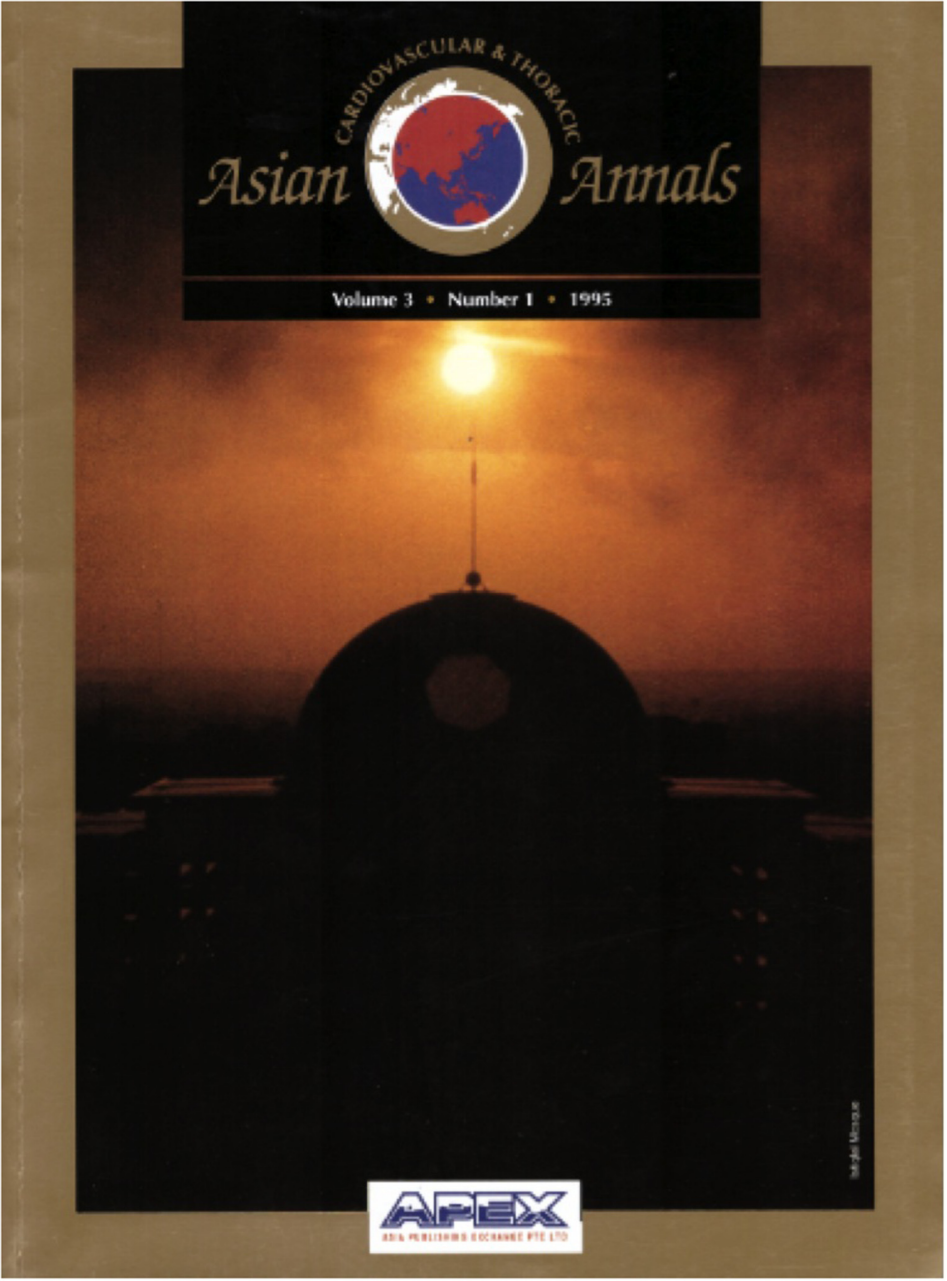
The first cover of the ‘Scenes from Asia’ series. The Istiqlal Mosque, Jakarta (Indonesia).

A major format change was the initiation of the ‘Scenes from Asia’ series, starting in 1995 (Figure 5). For over, a decade readership was presented with a cover depicting a historical landmark or scenic representation from Asia. The Publisher intended to draw readers’ attention to the striking geographical vastness of Asia and its advancements in cardiovascular medicine. The ‘Regional News’ section in each issue informed readers about new open-heart programmes, visits by foreign delegations, regional conferences, and the latest technology through the journal pages, just as APEX Newsletter had done between 1985 and 1992. Readers could gain a better understanding of the diverse components to the impressive progress in Asian healthcare at the time.

The photographs were taken by the publisher, *Asian Annals* staff, editors, colleagues and friends who kindly submitted photos to the Editorial Office in Singapore. The main issue with the ‘Scenes from Asia’ covers was the printing costs, which entailed multiple colours and high-quality printing paper. A complex process but one had to be distinguished the journal from all others. Further details on the start of *Asian Annals* are available also online.^27^

**Figure 6.**
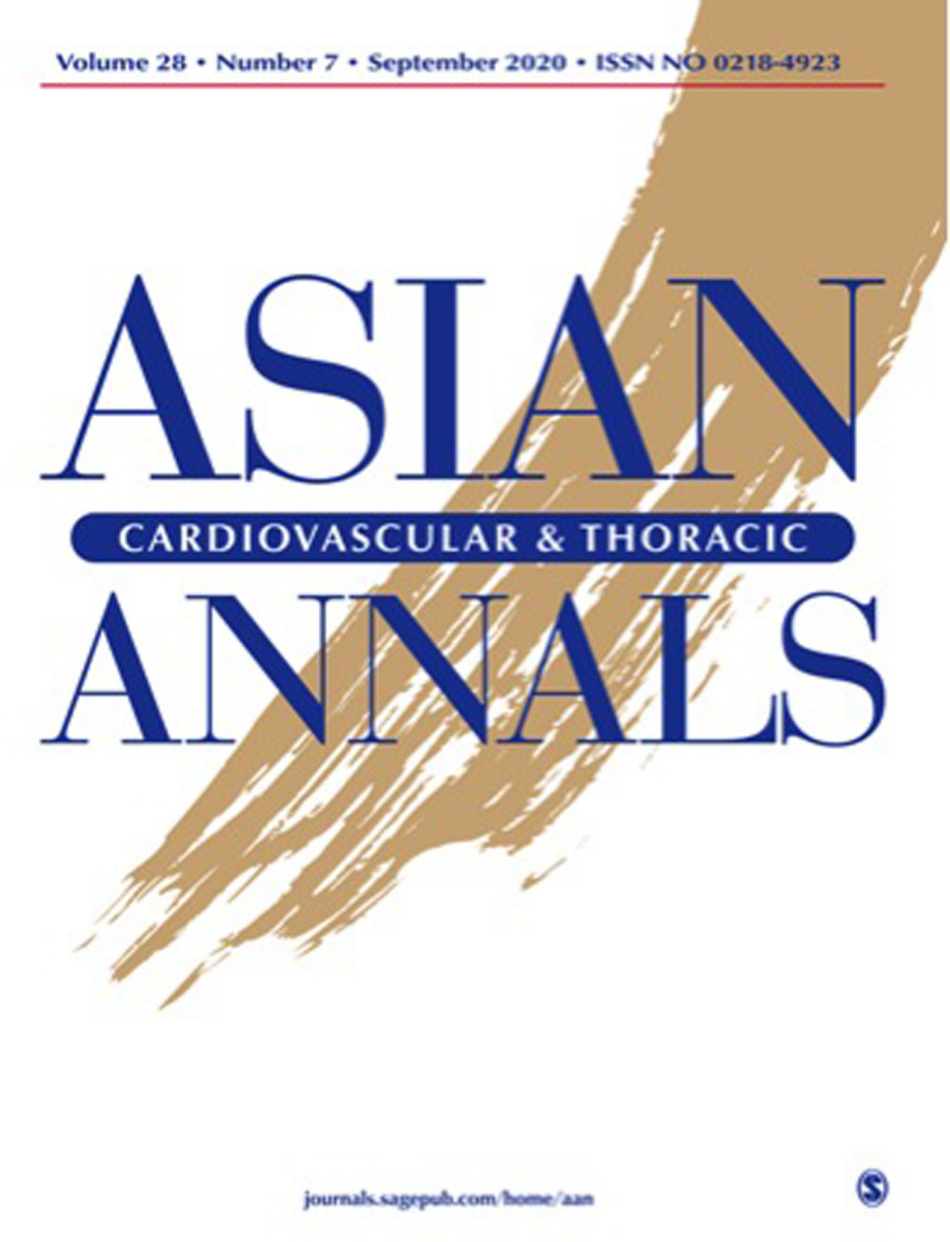
Current cover of the *Asian Cardiovascular and Thoracic Annals*.

Starting on the first issue of 2004, the cover was printed with white background and the name of *Asian Annals* in light blue capital letters in the middle of the page crossed by an ocher brush stroke (Figure 6). This has remained unchanged until today.

## Contents adaptability

*Asian Annals* is a member of the Committee for Publication Ethics (COPE)^2^ and fully adheres to publication ethics.^28^*Asian Annals* follows the IMRAD style^29,30^ in line with any other modern publication. And, of course, the universally accepted original articles, case reports, images, review articles, editorials and editorial commentaries, and technical sections have been contemplated over these three decades.

## Key milestones and steps forward

The Editors-in-Chief understand that the first milestone of *Asian Annals* has been receiving continuous help over three decades from the different Associate/Section Editors, successive prominent members of the Editorial Board and a large contingent of altruistic reviewers that helped *Asian Annals* to improve quality over time. This cannot go unnoticed. The issue of quality was addressed by EIC Dr Cho^14^ and it was always the policy of *Asian Annals* taking reviewer comments very seriously as it has to be.

Another milestone has been the relationship with *Asian Annals* publishers. As known, Frank Tamru was the Founder and Publisher for its first decade. The enterpreneurial creation of APEX, based first in Hawaii and moved to Singapore in 1986, laid the groundwork for the launch of *Asian Annals*. Mrs. Patrica Siow, hired by Tamru for an unrelated project, was the Managing Editor until the office was relocated to Hong Kong. During these years, APEX was assisted by Silent Partners in Austin, Texas, USA. In Hong Kong, Dr David Cheung had invested his funds to manage *Asian Annals* and transferred to SAGE. SAGE owns *Asian Annals* and has an affiliation with the ASCVTS.

Having said that, some milestones have modeled *Asian Annals* into what is today, an Asian Journal with international projection beyond the extensive regional limits. They can be summarized as follows:

1. Despite significant shortcomings in terms of managing staff and limited budget, in 1998, *Asian Annals* was indexed/abstracted in the EMBASE/Excerpta Medica database, which allowed authors around the world to access our published papers.
2. In March 2001, *Asian Annals* joined the *Annals of Thoracic Surgery*, *The Journal of Thoracic and Cardiovascular Surgery* and the *European Journal of Cardio-Thoracic Surgery* on the Cardiothoracic Surgery Network (CTSNet) with full-text online via a simple click on www.ctsnet.org. Having *Asian Annals* placed side-by-side with these publications greatly encouraged the publisher and editorial board members to continue. The perceived value of being on CTSNet transformed into a reality and translated into a three-fold increase in the number of submissions/year immediately after being posted on CTSNet. Being on the Internet via CTSNet allowed *Asian Annals* to fully represent the interests of Asian cardiothoracic surgeons in what was quickly becoming a worldwide international network of increasing value with an estimated current membership in the range of 40,000 worldwide. *Asian Annals* owes a debt of gratitude to some prestigious individuals who supported us in this endeavour. Still, we are particularly grateful to the late Dr Thomas B. Ferguson (1923–2013), past president of the STS and the AATS for his continuous advice and guidance even before *Asian Annals* was launched.^31^ This was a long and difficult process.
3. After several failed attempts, in March 2002 *Asian Annals* was accepted for MEDLINE Indexing by the prestigious National Library of Medicine in the United States of America. This was a major scientific milestone and encouraged our contributors and readership to submit more scientific production aiming at increase its quality.
4. *The Asian Society of Cardiovascular and Thoracic Surgery* (ASCVTS). Incidentally, the ASCVTS was founded in 1993 as *Asian Society of Cardiovascular Surgery* (ASCVS), the same as *Asian Annals* was launched. The parent organization of the ASCVS was the Asian Chapter of *International Society of Cardiovascular Surgery* (ISCVS), created in 1972, which changed its name to *Asian Society for Cardiovascular Surgery* (ASCVS) in 1993. In 2008, it turned into the current ASCVTS.^32^ The brief historical summary of Furuse^33^ is an elegant description of the sequence of events until ties were strengthened between the then ASCVS and *Asian Annals*. Early on, Frank Tamru sought a close relationship with ASCVTS as activity from their annual meeting was highlighted in the Regional News section, and several members already served on the *Asian Annals* Editorial Board. The direct affiliation between ASCVS and *Asian Annals* had to wait until the year 2000 when *Asian Annals* became the official publication of ASCVS, later ASCVTS, a relationship that lasts until today and will hopefully last forever.
5. The year 2006 witnessed the transition from four to six issues, which is the minimum to aspire to be recognized as a major international journal and eventually get an impact factor. And, in the year 2014, *Asian Annals* transitioned to nine issues.
6. Transition to electronic manuscript processing started after 2008. This represented also a major step forward to improve efficiency and adapt to modern manuscript handling.
7. In January 2021, *Asian Annals* became an online-only journal. This is a trend in the current publishing business. Online-only has superior benefits versus printed publications, including immediate access to recently published articles. This latest step of *Asian Annals* is in line with other prominent publications in the field,^34^ which have readapted to cope with changes over time and new demands of the readership.

## Conclusions

*The Asian Cardiovascular and Thoracic Annals*, the official journal of the *Asian Society for Cardiovascular and Thoracic Surgery*, is a young publication that has reached maturity. However, the process has been bumpy, and instability was sometimes a concern. Structural changes and editorial policies observed and implemented during these three decades have made *Asian Annals* progress to a level of international recognition, making us, readers, staff and editors proud of what has been done until now. Everything began with an enterprising American devoting his career to Asia and someone able to overcome roadblocks and produce the region's first scientific journal covering surgical treatment and the care for the heart.

The future starts today and *Asian Annals* will continue to move forward maintaining the highest standards of quality. This depends, of course, of readers and contributors submitting the best of their work. Thank you!
